# Improving assessment of lesions in longitudinal CT scans: a bi-institutional reader study on an AI-assisted registration and volumetric segmentation workflow

**DOI:** 10.1007/s11548-024-03181-4

**Published:** 2024-05-30

**Authors:** Alessa Hering, Max Westphal, Annika Gerken, Haidara Almansour, Michael Maurer, Benjamin Geisler, Temke Kohlbrandt, Thomas Eigentler, Teresa Amaral, Nikolas Lessmann, Sergios Gatidis, Horst Hahn, Konstantin Nikolaou, Ahmed Othman, Jan Moltz, Felix Peisen

**Affiliations:** 1 Fraunhofer Institute for Digital Medicine MEVIS, Bremen, Germany; 2 Diagnostic Image Analysis Group, Radboudumc, Nijmegen, Netherlands; 3 Department of Diagnostic and Interventional Radiology, Tübingen University Hospital, Eberhard Karls University, Tübingen, Germany; 4 Radiologisches Zentrum Offenbach-Dietzenbach, Dietzenbach, Germany; 5 Department of Dermatology, Center of Dermato-Oncology, Tübingen University Hospital, Eberhard Karls University, Tübingen, Germany; 6 Department of Dermatology, Venereology and Allergology, Charité University Hospital Berlin, Berlin, Germany; 7 Max Planck Institute for Intelligent Systems, Tübingen, Germany; 8 Cluster of Excellence iFIT (EXC 2180) “Image-Guided and Functionally Instructed Tumor Therapies”, Faculty of Medicine, Eberhard Karls University, Tübingen, Germany; 9 Institute of Neuroradiology, Johannes Gutenberg University Hospital Mainz, Mainz, Germany

**Keywords:** AI-assisted reading, Longitudinal CT scans, Image registration, Lesion segmentation, Oncology

## Abstract

**Purpose:**

AI-assisted techniques for lesion registration and segmentation have the potential to make CT-based tumor follow-up assessment faster and less reader-dependent. However, empirical evidence on the advantages of AI-assisted volumetric segmentation for lymph node and soft tissue metastases in follow-up CT scans is lacking. The aim of this study was to assess the efficiency, quality, and inter-reader variability of an AI-assisted workflow for volumetric segmentation of lymph node and soft tissue metastases in follow-up CT scans. Three hypotheses were tested: (H1) Assessment time for follow-up lesion segmentation is reduced using an AI-assisted workflow. (H2) The quality of the AI-assisted segmentation is non-inferior to the quality of fully manual segmentation. (H3) The inter-reader variability of the resulting segmentations is reduced with AI assistance.

**Materials and methods:**

The study retrospectively analyzed 126 lymph nodes and 135 soft tissue metastases from 55 patients with stage IV melanoma. Three radiologists from two institutions performed both AI-assisted and manual segmentation, and the results were statistically analyzed and compared to a manual segmentation reference standard.

**Results:**

AI-assisted segmentation reduced user interaction time significantly by 33% (222 s vs. 336 s), achieved similar Dice scores (0.80–0.84 vs. 0.81–0.82) and decreased inter-reader variability (median Dice 0.85–1.0 vs. 0.80–0.82; ICC 0.84 vs. 0.80), compared to manual segmentation.

**Conclusion:**

The findings of this study support the use of AI-assisted registration and volumetric segmentation for lymph node and soft tissue metastases in follow-up CT scans. The AI-assisted workflow achieved significant time savings, similar segmentation quality, and reduced inter-reader variability compared to manual segmentation.

**Supplementary Information:**

The online version contains supplementary material available at 10.1007/s11548-024-03181-4.

## Introduction

**Fig. 1:**
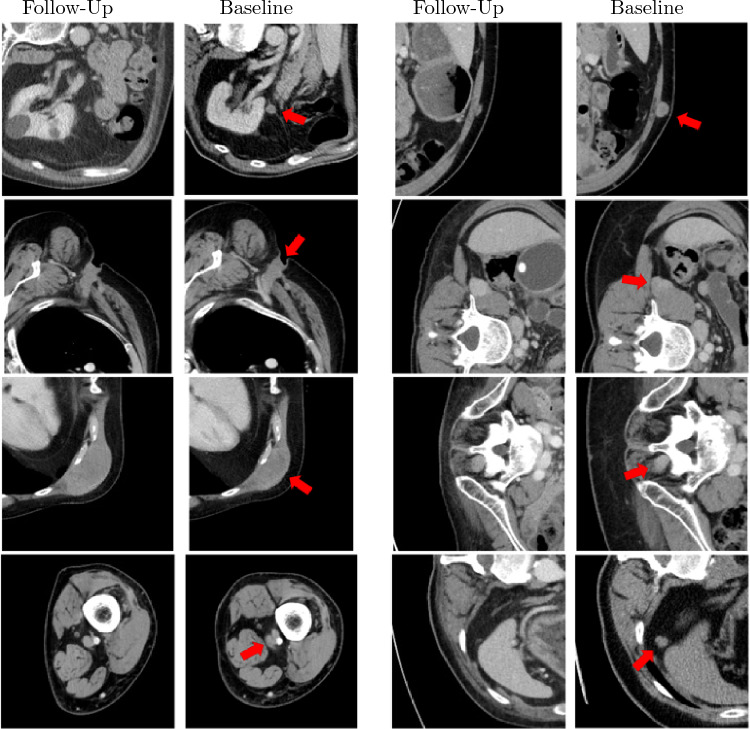
Examples of soft tissue lesions at different locations. The first and third rows show the lesion in the baseline scan and the second and fourth row show the lesion in the corresponding follow-up scan

The measurement of metastatic tumors using longitudinal computer tomography (CT) scans is crucial for evaluating the efficacy of cancer treatment. However, manual measurements based on the diameter of lesions using Response Evaluation Criteria in Solid Tumors (RECIST) criteria [1] can be time-consuming and error-prone. Moreover, RECIST criteria are limited in their ability to capture tumor heterogeneity and do not account for changes in the overall tumor burden. Previous studies have demonstrated that volumetric artificial intelligence (AI)-assisted segmentation can deliver additional tumor information, such as volumetric RECIST and total tumor volume, and has the potential to provide more accurate assessments of treatment response than diameter-based RECIST [2, 3]. Therefore, the development of lesion segmentation algorithms based on artificial intelligence has the potential to significantly improve response evaluation by enabling automated and accurate tumor measurements and therefore help to handle the ever-growing mass of image-based staging and follow-up evaluation [4]. Additionally, in the field of radiomics, the extraction of multiple quantitative features from segmented structures in medical images [5] resulting in the conversion of medical images into minable data and the subsequent analysis promises new insights into therapy response and hold the potential to revolutionize medical image-based evaluation techniques [6]. Both fields have a huge clinical impact and share a common requirement: an accurate lesion segmentation, obtained with minimal manual effort. U-Nets are one of the current state-of-the-art approaches in deep learning and an established and preferred method for image segmentation [7–9]. While there are many successful applications for organs such as the liver [10], only a few studies investigated the segmentation of other lesions, such as lymph node metastases [11]. To our knowledge, no study has evaluated the application to soft tissue metastases to date. Soft tissue metastases are very common in melanoma patients, but they provide a particular challenge for image evaluation as they can arise in a variety of locations (cutaneous, subcutaneous, muscular, retroperitoneal) and shapes (round, multilobular, well-defined, invasive; see Fig. 1). They are often primarily small and, if not surrounded by fatty tissue, extremely hard to distinguish. The present reader study evaluates the practical application of a recently introduced U-Net-based pipeline [12] for automated registration and volumetric segmentation of soft tissue metastases in follow-up CTs, which has meanwhile been extended to lymph node segmentation. While the previous publication contained a technical validation, this paper conducted a bi-institutional reader study to assess the efficacy and applicability of the proposed AI-assisted workflow for lymph node and soft tissue metastases in follow-up CTs. The detection of new metastases was not the scope of the present study. Thus, the three hypotheses of the study were:

### (H1)

Assessment time for follow-up lesion segmentation is reduced using an AI-assisted workflow.

### (H2)

The quality of AI-assisted segmentation is non-inferior to the quality of fully manual segmentation.

### (H3)

The inter-reader variability of the resulting segmentations is reduced with AI assistance.

## Material and methods

### Study design and subjects

The study utilized the Central Malignant Melanoma Registry in Germany (CMMR) to retrospectively identify patients diagnosed with stage IV melanoma between 2015-01-01 and 2018-12-31 who were initially treated at the Department of Dermatology, University Hospital Tübingen which is a tertiary referral center for melanoma patients. The study protocol was approved by the institutional ethics board, and informed consent was waived due to the retrospective study design. 272 patients who met the inclusion criteria (stage IV melanoma, baseline (pre-treatment) contrast-enhanced CT, lymph node and/or soft tissue metastasis at baseline CT, available first follow- up CT after therapy initiation), were selected. CTs of these patients, with various types of soft tissue and lymph node metastases in baseline and follow-up CTs, were divided into a training and validation set for developing the pipeline [12], and a testing set for the present study. The metastases were radiologically identified by morphological criteria or their behavior under therapy in the follow-up CTs. The training, validation, and reference segmentations for the testing set were manually conducted by an experienced resident radiologist (FP 4 years) in consensus reading with two senior radiologists with extensive experience in oncologic imaging (AO 8 years and SG 9 years) using a custom-made reading software (SATORI; Fraunhofer MEVIS, Bremen). Figure 1 shows exemplary soft tissue metastases used in our study. In the appendix, Figure A1 shows the detailed study workflow, Table B1 shows the patient characteristics and Table B2 lists the scanner types and number of scans acquired with the respective scanner.

### Training and validation dataset

The training and validation set included 4308 lesions (2603 soft tissue and 1705 lymph node lesions) from 214 patients split into 3445 (2081 soft tissue and 1364 lymph node lesions) and 863 (522 soft tissue and 341 lymph node lesions) lesions for training and validation, respectively. The datasets included cases from different institutions and were therefore obtained with different CT scanners with various protocols. Typical CT imaging parameters used in our center for staging of melanoma patients are reported in the appendix Table C1.

### Testing dataset

The testing dataset included 126 soft tissue and 135 lymph node lesions from 58 patients referred for the first follow-up CT after therapy started that were not included in the training and validation dataset. We selected patients with lower lesion counts for the testing set to obtain a diverse set of lesions. For details refer to Table B1 and B2. The lesions were stratified by diameter size in the follow-up scan as smaller than 10 mm (n = 58), 10–20 mm (n = 94), and larger than 20 mm in diameter (n = 55), with a mean size of 17.9 mm ± 15.2 mm (range: 5.0–140.5 mm). 54 lesions showed complete response.

### Manual and AI-assisted study workflow

**Fig. 2:**
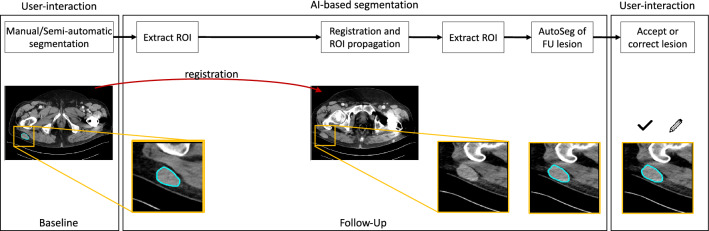
Schema of the proposed pipeline for AI-assisted segmentation of lymph node and soft tissue metastases in follow-up CT scans. The AI-assisted segmentation pipeline includes four major components: 1.) Extraction of the region of interest (ROI) around the lesion in the baseline scan; 2.) Registration of the baseline to the follow-up scan; 3.) Propagation of the ROI to the follow-up scan to constrain the search region. Inference of the trained U-Net to segment all the lesions in the defined region; 4) Selection of the corresponding lesion from the output of the U-Net. This reader study focuses on the user-interaction on the follow-up image

Readers viewed baseline and follow-up CTs simultaneously in a single window using the custom-made reading software. Manual segmentation involved outlining lesions on the follow-up CT images using a cursor with optional interpolation for neighboring slices. The AI-assisted workflow used automatically generated volumetric segmentations that were displayed in SATORI (see Figure G1 in appendix). The readers had the option to accept the automated segmentation as perfect, accept it as passable and make manual corrections, or dismiss it and perform manual segmentation. If the AI produced a segmentation for metastases showing a complete response in follow-up CTs, radiologists could reject the proposed structure and save an empty structure. A schematic representation of the proposed pipeline is presented in Fig. 2. Extensive technical details have been published in a previous publication [12] and are summarized in the appendix D. Three radiologists from two institutions independently segmented the testing set to assess inter-reader agreement variability. The radiologists were reader 1 (MM, specialist, Tübingen), reader 2 (HA, resident, Tübingen) and reader 3 (BG, physician, Bremen) with 7, 2, and 5 of experience in oncologic radiology, respectively.

The readers manually segmented the first 50% of the testing cohort, followed by AI-assisted segmentation of the second 50%. After two weeks, they performed AI-assisted segmentation of the first 50% and manual segmentation of the second 50% of the cohort to avoid recall bias. To prevent an artificial habituation effect, patients were sorted by ID and not by the number of metastases. The readers were blinded to their previous segmentation results, those of other readers, and the reference standard of the follow-up examinations. In the following, *Mi* and *Ai* denote the manual session and the assisted session of the reader *i* with *i* ∈ {1, 2, 3}.

## Experiments

The experimental analysis was based on the three hypotheses. Therefore, we evaluated reading time, accuracy, and inter-reader variability.

### Reading time

User interaction time was recorded for manual segmentations, as well as for verification and manual corrections of automated segmentations per patient. A digital timer, included in the reading software interface, was manually started by each reader for each patient before starting the segmentation and manually stopped after finishing the segmentation of the respective patient.

### Accuracy

The study evaluated the detection and segmentation of lesions in follow-up CTs, given a baseline lesion segmentation for each combination of reader and availability of AI support. Lesions could either still be present in the follow-up scan or disappear under therapy. The detection performance was evaluated for each lesion and session against the reference standard with the following categories:

- True positive (TP): lesion annotated both by the reference reader and in the evaluated session.
- False negative (FN): lesion annotated by the reference reader but marked as disappeared in the evaluated session.
- False positive (FP): lesion marked as disappeared by the reference reader but annotated in the evaluated session.
- True negative (TN): lesion marked as disappeared both by the reference reader and in the evaluated session. Furthermore, the sensitivity (TP/(TP + FN)) for lesions < 10 mm, 10–20 mm and > 20 mm and all lesions were calculated per session. Segmentation accuracy was evaluated against the reference standard and assessed by using the Dice score for all lesions that remained visible in the follow-up scan. The average symmetric surface distance was evaluated for all detected lesions in each session.

### Inter-reader variability

The Dice score was used to evaluate the inter-reader variability and additionally the inter-method variability of the lesion segmentations, respectively.

Inter-reader agreement of manual and AI-assisted volumetric lesion segmentation was evaluated with intraclass correlation coefficients (IBM SPSS Statistics 26), with a two-way random effects model, based on a mean-rating (*k* = 4) and absolute agreement definition selected, comparing the reference segmentation and the manual/AI-assisted segmentations by the three readers, respectively. The ICC values are interpreted as follows: < 0.5 poor, 0.5–0.75 moderate, 0.75–0.9 good and > 0.90 excellent reliability [13, 14].

### Statistical analysis

The statistical analysis primarily targeted two (co-primary) endpoints: reading time (seconds) and segmentation accuracy (Dice score), comparing the assisted to the manual workflow. We considered an average Dice score loss of up to 0.05 as non-inferior. For both analyses, a Bayesian mixed-effects generalized linear model was fit with the statistical software R (version 4.2.1) and the brms package (version 2.18.0). Adding appropriate random effects (e.g. reader, lesion within patient) to the model allowed us to generalize our findings to unknown lesions, patients and readers. In a secondary analysis, inter-reader agreement was assessed by modeling the Dice score in each pair of readers in the study. A more detailed description is given in the appendix E.

## Results

Lymph nodes and soft tissue metastases share common morphological properties. Both lesion types do not arise in parenchymatous organs or the central nervous system, are often surrounded by fatty tissue or develop in close relationship to structures such as vessels, muscular tissue, or intestine. Despite predefined regions, lymph node metastases can, likewise soft tissue metastases, unfold in almost any region of the body [15]. Therefore, the analysis for the two entities was grouped and results are not reported separately.

### Reading time

Mean interaction time was 342 s ± 426 per patient for manual segmentation and 222 s ± 312 s per patient for AI-assisted segmentation (M1: 204 s ± 288 s; M2: 222 s ± 318 s; M3: 594 s ± 516 s; A1: 102 s ± 108; A2: 84 s ± 84; A3: 474 s ± 414 s).

### Accuracy

The detection performance and sensitivity for the manual and AI-assisted workflow are summarized in Table 1.

**Table 1:** Detection and sensitivity performance. Segmentation performed manual (M) and AI-assisted (A) by reader 1 (MM), 2 (HA), and 3 (BG)

Method	User	TP	FN	FP	TN	Sensitivity			
						all	< 10 mm	10–20 mm	> 20 mm
Manual	M1 (MM)	192	15	8	46	0.93 (0.88,0.96)	0.88 (0.77,0.94)	0.94 (0.87,0.97)	0.96 (0.88,0.99)
	M2 (HA)	190	17	8	46	0.92 (0.87,0.95)	0.86 (0.75,0.93)	0.91 (0.84,0.96)	0.98 (0.9,1.0)
	M3 (BG)	172	35	23	31	0.83 (0.77,0.88)	0.78 (0.65,0.86)	0.86 (0.78,0.92)	0.84 (0.72,0.91)
Assisted	A1 (MM)	198	9	10	44	0.96 (0.92,0.98)	0.93 (0.84,0.97)	0.95 (0.88,0.98)	1.0 (0.93,1.0)
	A2 (HA)	193	14	11	43	0.93 (0.89,0.96)	0.91 (0.81,0.96)	0.93 (0.85,0.96)	0.96 (0.88,0.99)
	A3 (BG)	178	29	23	31	0.86 (0.81,0.9)	0.84 (0.73, 0.92)	0.86 (0.78,0.92)	0.87 (0.76,0.94)

*TP* true positive, *FN* false negative, *FP* false positive, *TN* true negative

Data in parentheses are 95% confidence intervals. The results are differentiated by the diameter of the reference segmentation smaller than 10 mm, 10–20 mm and larger than 20 mm

**Fig. 3:**
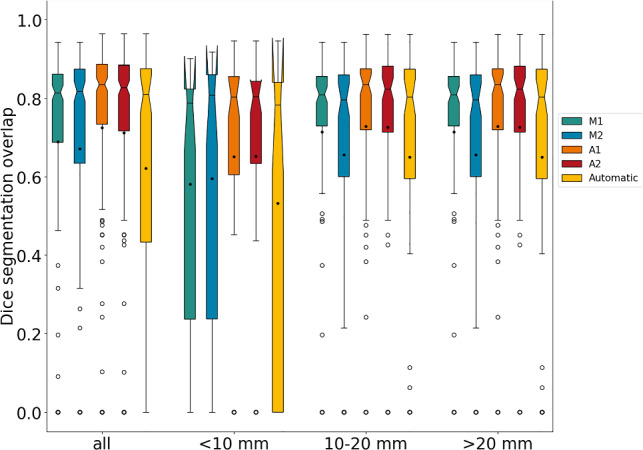
Boxplots of the Dice score for manual segmentations (M1, M2 and M3) and AI-assisted segmentation (A1, A2 and A3) evaluated against the reference standard split by the diameter of the reference segmentation (< 10 mm, 10–20 mm, > 20 mm). The mean is symbolized by black dots, median by black horizontal lines

**Fig. 4:**
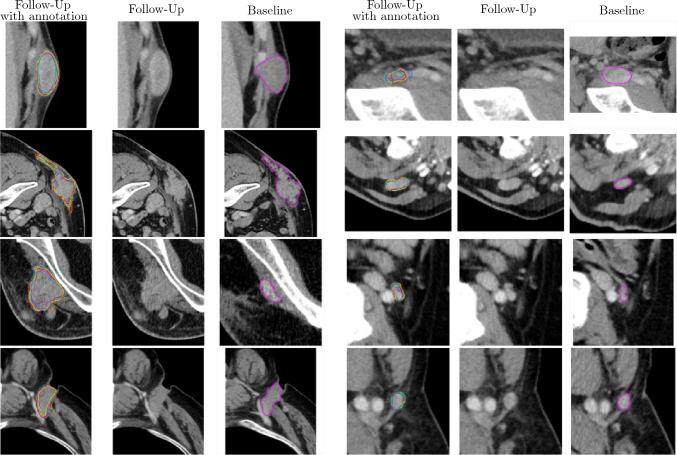
Exemplary segmentation results for the reference reader (pink), the manual segmentation M1 (green), M2 (blue), M3 (cyan) and the assisted segmentation A1 (orange), A2 (red) and A3 (yellow). In some cases, the assisted segmentation of readers is the same and therefore not fully shown

The sensitivity was lower for smaller lesions compared to larger lesions across all readers. The manual and assisted sessions yielded good scores for all readers, with reader 1 and reader 2 achieving slightly better scores than reader 3. Table 2 presents an overview of the segmentation results, which are also depicted in Fig. 3. The median Dice scores of manual and AI-assisted segmentations were 0.81–0.82, and 0.80–0.84 indicating comparable performance. The Dice score was slightly lower for small lesions (< 10 mm) compared to larger lesions. Exemplary segmentation results are illustrated in Fig. 4.

**Table 2:** Segmentation performance. Median Dice score and average surface distance. 25%- and 75% quantile in parentheses

Method	User	Dice score				Average surface distance (mm)			
		all	< 10 mm	10–20 mm	20 mm	all	< 10 mm	10–20 mm	> 20 mm
Manual	M1 (MM)	0.82 [0.71,0.86]	0.79 [0.47,0.83]	0.81 [0.73,0.86]	0.85 [0.79,0.89]	0.4 [0.3,1.0]	0.3 [0.2,0.5]	0.5 [0.3,0.9]	0.7 [0.4,1.2]
	M2 (HA)	0.82 [0.67,0.87]	0.81 [0.35,0.86]	0.8 [0.6,0.86]	0.84 [0.76,0.9]	0.5 [0.3,1.3]	0.2 [0.2,1.1]	0.5 [0.3,1.0]	0.8 [0.4,1.4]
	M3 (BG)	0.81 [0.69,0.86]	0.78 [0.58,0.83]	0.8 [0.72,0.85]	0.85 [0.76,0.88]	0.5 [0.3,1.0]	0.4 [0.2,1.1]	0.5 [0.3,0.9]	0.6 [0.4,1.1]
Assisted	A1 (MM)	0.84 [0.74,0.89]	0.81 [0.62,0.86]	0.84 [0.72,0.88]	0.87 [0.8,0.91]	0.4 [0.2,0.9]	0.3 [0.2,0.8]	0.5 [0.2,0.8]	0.5 [0.3,0.9]
	A2 (HA)	0.83 [0.72,0.89]	0.81 [0.67,0.84]	0.82 [0.71,0.88]	0.86 [0.79,0.9]	0.4 [0.2,0.9]	0.3 [0.2,0.6]	0.5 [0.2,0.8]	0.7 [0.3,1.2]
	A3 (BG)	0.8 [0.67,0.88]	0.76 [0.64,0.83]	0.8 [0.66,0.87	0.83 [0.77,0.9]	0.4 [0.1,0.8]	0.3 [0.1,0.6]	0.4 [0.2,0.9]	0.5 [0.1,0.9]

The results are split by the diameter of the reference segmentation (< 10 mm, 10–20 mm, > 20 mm)

### Inter-reader variability

Table 3 presents a summary of the inter-reader variability and inter-method variability using the Dice score between the corresponding segmentations. The median Dice scores for segmentations generated by manual annotation ranged from 0.80 to 0.82. The assisted segmentations achieved higher median Dice scores of 0.85–1.0. For two of the readers (reader 1 and 2), the median Dice score in the AI-assisted session was 1.0, indicating that in more than 50% of the lesions, the readers accepted the segmentation suggested by AI without any further corrections.

**Table 3:** Inter-reader and inter-method agreement displayed by the Dice score computed between the segmentations generated with the respective methods

Method	Reference	M1	M2	M3	A1	A2	A3
Reference		0.82 [0.71, 0.86]	0.82 [0.67, 0.87]	0.81 [0.69, 0.86]	0.84 [0.74, 0.89]	0.83 [0.72, 0.89]	0.8 [0.67, 0.88]
M1	0.82 [0.71, 0.86]		0.82 [0.68, 0.87]	0.8 [0.66, 0.85]	0.83 [0.72, 0.87]	0.81 [0.68, 0.87]	0.79 [0.61, 0.85]
M2	0.82 [0.67, 0.87]	0.82 [0.68, 0.87]		0.8 [0.59, 0.85]	0.83 [0.69, 0.88]	0.82 [0.59, 0.87]	0.79 [0.6, 0.86]
M3	0.81 [0.69, 0.86]	0.8 [0.66, 0.85]	0.8 [0.59, 0.85]		0.81 [0.66, 0.87]	0.82 [0.7, 0.87]	0.8 [0.69, 0.85]
A1	0.84 [0.74, 0.89]	0.83 [0.72, 0.87]	0.83 [0.69, 0.88]	0.81 [0.66, 0.87]		1.0 [0.88, 1.0]	0.86 [0.72, 0.92]
A2	0.83 [0.72, 0.89]	0.81[0.66, 0.87]	0.82 [0.59, 0.87]	0.82 [0.7, 0.87]	1.0 [0.88, 1.0]		0.85 [0.7,0.92]
A3	0.8 [0.67, 0.88]	0.79 [0.61, 0.85]	0.79 [0.6, 0.86]	0.8 [0.69, 0.85]	0.86 [0.72, 0.92]	0.85 [0.7, 0.92]	

The median and the 25%- and 75% quantile in parentheses are reported

Inter-reader agreement was good for manual segmentations (overall ICC 0.80). When the lesions were analyzed split by size (< 10 mm, 10–20 mm and > 20 mm) agreement of manual volumetric segmentations was poor for lesions < 10 mm (ICC 0.31) and good for lesions 10-20 mm (ICC 0.90) and < 20 mm (ICC 0.82). AI-assisted segmentation improved the inter-reader agreement (overall ICC 0.84). The effect was especially present for lesions < 10 mm (ICC 0.77). For detailed values see Table F1 in the appendix.

### Statistical analysis

There is very strong evidence that the assisted workflow is faster compared to the manual workflow. The expected marginal effect (assisted—manual) for an unknown reader is estimated to be − 169.4 s (median: − 83.6; 95% CI [− 704.521, − 3.4]). The (posterior) probability of an effect size below zero is estimated to be 0.983. Regarding accuracy, there is very strong evidence for a non-inferior Dice score of the assisted workflow compared to the manual workflow.

The expected marginal effect (assisted—manual) for an unknown reader is estimated to be 0.015 (median: − 0.011; 95% CI [− 0.061, 0.115). The (posterior) probability of non-inferiority, i.e., an effect size larger than the non-inferiority margin − 0.05 is estimated to be 0.968. Inter-reader agreement was measured via the Dice score between annotations (by three different readers) for each lesion. The expected marginal effect (assisted—manual) for an unknown pair of readers is 0.086 (median: 0.063; 95% CI [− 0.141, 0.486]). Thus, we have modest evidence that the assisted workflow leads to higher inter-reader agreement compared to the manual workflow. The (posterior) probability for this posthoc hypothesis is estimated to be 0.825.

## Discussion

The study’s purpose was to evaluate the practical application of an AI-assisted registration and volumetric segmentation pipeline for lymph node and soft tissue metastases in follow-up CTs. All three hypotheses could be confirmed. (H1) The results of the study provided compelling evidence for the efficacy of the AI-assisted workflow, which was found to be significantly faster than the conventional manual workflow. The mean reading time required for volumetric lesion segmentation was substantially reduced by a third per patient using the proposed AI-assisted segmentation pipeline. (H2) Average segmentation quality was comparable in the AI-assisted and the manual workflow. The effect was present for all three categories of lesion size. The results even suggest that the quality of the segmentations for small lesions is better for the AI-assisted segmentations than the manual segmentations when computing the Dice or ASD compared to the reference reader. However, these results were not statistically analyzed and there is a slight bias because the training data was also annotated by the reference reader, so the AI might have adopted this annotation style. (H3) Inter-reader variability of volumetric annotations is reduced by using the AI-assisted workflow. The inter-reader agreement for the AI-assisted annotations was significantly higher than for manual annotations. Over 50% of the segmentation propositions in the AI assisted workflow were accepted with no further corrections by two of three radiologists. Additional intraclass correlation analyses revealed that there was a good inter-reader agreement for manual segmentations, which improved when adding AI-assistance. This was especially the case for small lesions < 10 mm. The fact that the pipeline reduces inter-reader variability is an important finding. Resulting segmentations become more comparable between different readers, making the transferability of resulting parameters such as tumor volume or diameters easier and more reader independent. The statistical analysis conducted in this study allows for a generalization of our findings to new patients and readers. The reduction of reading time, non-inferiority of the segmentation quality, and a decrease in inter-reader variability can be transferred to new patients and readers with similar characteristics to those examined in this study. The observations presented in this study are consistent with previous studies such as the work by Vorontsov et al., which reported similar effects for the correction of fully automated segmentation of liver lesions in CTs of patients with colorectal cancer liver metastases using a CNN [16]. Likewise, Moltz et al. evaluated a simpler algorithm for automatic lesion tracking and segmentation in follow-up CTs for lung nodules, liver metastases and lymph nodes and reported a reduction of assessment time through lesion tracking [17]. The acceleration of volumetric lesion segmentation is crucial for the translation of modern radiological techniques, such as radiomics, into clinical applications. However, to be accepted by the radiological community, an AI-assisted workflow must be non-inferior to a manual workflow. Therefore, the non-inferiority of the AI-assisted segmentation in this study is an important finding and is in line with a previous publication evaluating automated lesion tracking and segmentation of lung nodules, liver metastases and lymph nodes [17]. Lower Dice scores were especially present for small lesions < 10 mm. Vorontsov et al. [16] reported similar results. This can be explained by the fact that even deviations of a few voxels account for a large percentage in small lesions and user correction attenuates this effect, especially in small lesions. Although this study has a bi-institutional design and included a large sample of lesions, it is important to acknowledge its limitations. Ideally, even more patients and readers should have been included in the study to further validate the results and to capture inter-rater variability in a more reliable way, but this is an inherent limitation most reader studies share. However, the statistical analysis used in the study takes this into consideration, and the findings can be generalized to new patients, lesions, and readers with similar characteristics to those examined in this study. Another limitation of the study is that the baseline segmentation was not evaluated, which could have provided valuable information. Due to the fact that most patients received their baseline and follow-up imaging in one institution, there is a bias towards Siemens scanners. A potential alternative to manual baseline segmentation might be AI-assisted one-click segmentation [18] or fully automatic detection. The analysis is conservative in the sense that further training of the readers with the assisted workflow might lead to an additional improvement in one or both outcomes over time [19].

## Conclusion

The present reader study confirmed that AI-assisted CT follow-up registration and volumetric segmentation of lymph node and soft tissue metastases significantly reduced the reading time, as well as inter-reader variability with similar segmentation quality compared to manual segmentation. This has a huge clinical impact, as several radiological techniques, such as (volumetric) RECIST-measurements and radiomic analysis heavily rely on fast and accurate segmentations and new approaches are in demand to reduce manual effort. To our knowledge, no study has evaluated a volumetric application for soft tissue metastases to date. Our study closes this gap and describes an applicable volumetric segmentation pipeline that can easily be transferred to other lesion types in future investigations.

### Supplementary Information

Below is the link to the electronic supplementary material.

Supplementary file1 (PDF 323 kb)
