# Supplementary material for: Improving assessment of lesions in longitudinal CT scans: a bi-institutional reader study on an AI-assisted registration and volumetric segmentation workflow
Source: Int J Comput Assist Radiol Surg. 2024 May 30;19(9):1689–97. doi: 10.1007/s11548-024-03181-4 (PMC11365847; doi:10.1007/s11548-024-03181-4)
Supplement: Supplementary file 1 — Supplementary file1 (PDF 323 kb) [file 11548_2024_3181_MOESM1_ESM.pdf]

Electronic Supplementary Material

Improving assessment of lesions in longitudinal CT scans: A bi-institutional reader study on an AI-assisted registration and volumetric segmentation workflow

Alessa Hering, Max Westphal, Annika Gerken, Haidara Almansour, Michael Maurer, Benjamin Geisler, Temke Kohlbrandt, Thomas Eigentler, Teresa Amaral, Nikolas Lessmann, Sergios Gatidis, Horst Hahn, Konstantin Nikolaou, Ahmed Othman, Jan Moltz, Felix Peisen

Appendix A: Study workflow

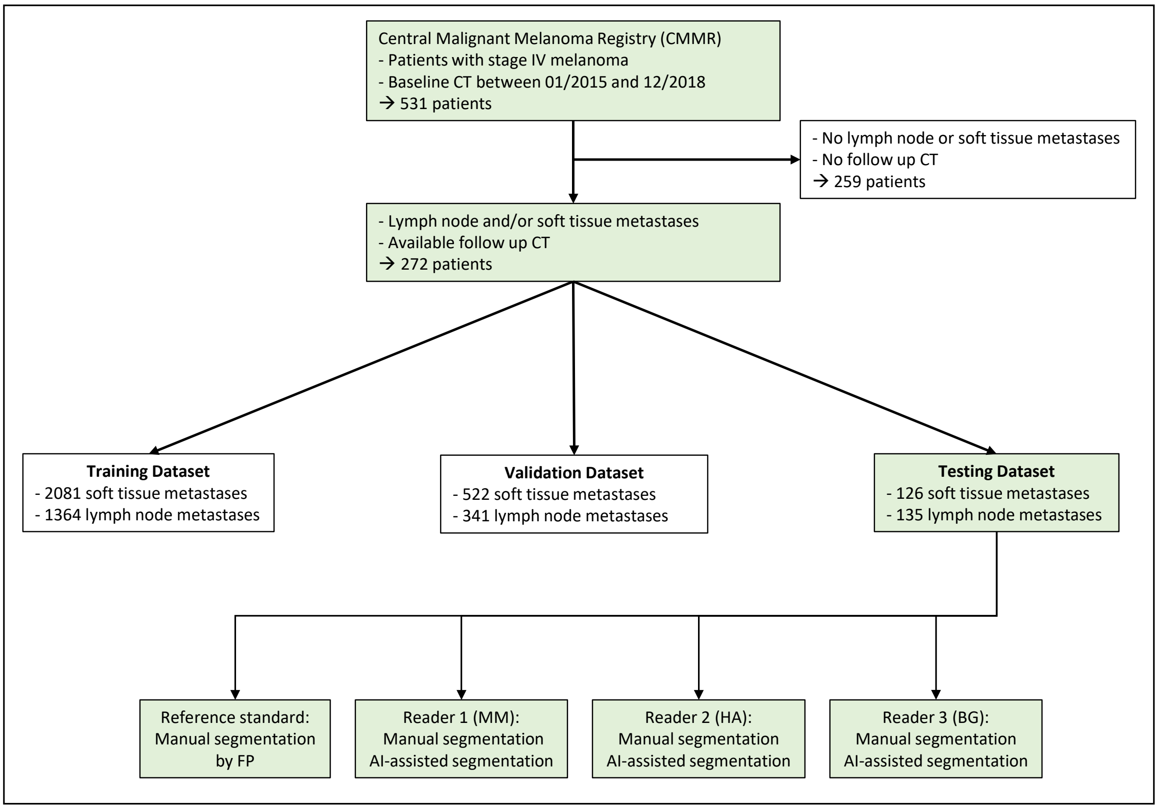

Figure A1: Study workflow. The green highlighted fields mark the different steps carried out for the present reader study and analysis.

Appendix B: Dataset characteristics

Table B1: Patient demographics and characteristics of the datasets.

	Total Dataset	Training + Validation Dataset	Testing Dataset
**Patients**	272	214	58
**Age (years, [SD])**	63.6 (14.6)	63.4 (14.9)	64.7 (13.2)
**Gender (female)**	44%	44%	37%
**Stage IV (AJCC 8th Edition)**	100%	100%	100%
**Treatment**			
Immunotherapy	72%	73%	67%
Targeted therapy	28%	27%	33%
**Number of lesions (total)**	4571	4308	263
Lymph node	1842	1705	135
Soft tissue	2729	2603	126
**Median number of lesions (per patient)**	7	10	5
Lymph node	3	4	3
Soft tissue	4	6	2
**Inhouse CT**	81%	78%	86%
**External CT**	19%	22%	14%

Table B2: List of different scanner types and number of scans acquired with that scanner type.

	scanner	vendor	number of patients		
			baseline	follow-up	total
**training and validation cohort**					
inhouse	SOMATOM Definition AS+	Siemens	46	47	93
	SOMATOM Definition Flash	Siemens	4	8	12
	SOMATOM Force	Siemens	82	109	191
	Sensation 64	Siemens	22	20	42
	Biograph 128	Siemens	28	24	52
external	Aquillion One	Canon	3		3
	Astelion	Canon	2	1	3
	Discovery 710	General Electrics	1		1
	LightSpeed VCT	General Electrics	1		1
	Optima CT540	General Electrics	1		1
	Optima CT660	General Electrics	1		1
	Briliance	Philips		1	1
	Ingenuity Core	Philips	1		1
	Biograph 128	Siemens	1		1
	Biograph 64	Siemens	3		3
	Emotion 16	Siemens	2	2	4
	Emotion 6	Siemens	2		2
	Perspective	Siemens	1		1
	Scope	Siemens	1	1	2
	Sensation 64	Siemens	3		3
	Sensation Cardiac	Siemens	1		1
	SOMATOM Definition AS	Siemens	5		5
	SOMATOM Definition Edge	Siemens	1		1
	SOMATOM Definition Flash	Siemens	1		1
	SOMATOM Force	Siemens	1	1	2
total			214	214	428
**testing cohort**					
inhouse	SOMATOM Definition AS+	Siemens	6	10	16
	SOMATOM Definition Flash	Siemens	3	1	4
	SOMATOM Force	Siemens	22	23	45
	Sensation 64	Siemens	5	7	12
	Biograph128	Siemens	11	11	22
external	Aquillion One	Canon	1	1	2
	Lightspeed VCT	GE	1		1
	Optima CT540	GE	1		1
	Ingenuity Core	Philips		1	1
	Biograph64	Siemens	1		1
	Emotion 16	Siemens	2	1	3
	Scope	Siemens		1	1
	SOMATOM Definition AS	Siemens	4	1	5
	SOMATOM Definition Edge	Siemens	1		1
	SOMATOM Definition Flash	Siemens		1	1
total			58	58	116

Appendix C: CT Parameters

Table C1: Inhouse standard CT parameters for melanoma whole body staging.

Reference current	240 mAs
Tube voltage	120 kV
Collimation	128 x 0.6 mm
Rotation time	0.5 s
Pitch	0.6
Image reconstruction	Medium smooth kernel
Contrast medium phase	Portal venous

Appendix D: Detailed description of the AI-assisted segmentation pipeline

Registration:

The registration must align the global structures but at the same time be locally accurate enough for precise lesion propagation. Therefore, we adopted a three-step approach to automatically register the baseline to the follow-up image: *1.)* Translational alignment; *2.)* Rigid registration; *3.)* Deformable registration. Hereby, the registration pipeline starts with robust methods with fewer degrees of freedom and moves on to more precise, but less robust methods, which require better starting points due to their higher degrees of freedom.

Translational alignment

The prealignment process in translation utilizes the FASTA (Fast Translation Alignment) technique, which involves a comprehensive grid search to calculate the Sum-of-Squared-Distances (SSD) across various possible translations. This method improves the precision of translation but requires more computational resources. To speed up the processing, the images being aligned (moving and fixed) are downscaled to a uniform resolution of 128x128x128. The sampling of the grid is adjusted to rates of 3, 3, and 51 along the x, y, and z axes, respectively. The primary focus of the prealignment is on z-translation, indicative of the typical central alignment of CT scans with the body's midpoint.

Rigid registration

The rigid registration uses the translational prealignment in z-direction as a starting point for a multi-level registration using the SSD distance measure. The method uses a Gauss-Newton optimization scheme to solve the optimization problem.

Deformable registration

The deformation is defined as a minimizer of the cost function

$$\min_{y} \mathcal{D}^{NGF}(\mathcal{F}, \mathcal{M}(y)) + \alpha \mathcal{R}^{curv}(y)$$

with the normalized gradient field distance measure $\mathcal{D}^{NGF}$ that focuses on the alignment of image gradients of the fixed image $\mathcal{F}$ and the deformed moving image $\mathcal{M}(y)$. The second-order curvature

regularizer $\mathcal{R}^{curv}$ enforces smooth deformation by penalizing spatial derivatives. The factor α serves as a weight in this equation. To address the optimization problem, the limited-memory Broyden-Fletcher-Goldfarb-Shannon (L-BFGS) optimization strategy is employed, integrated within a multi-level framework.

Lesion segmentation:

The registration was used to propagate the baseline mask to the follow-up scan. While this propagated mask may not be accurate enough due to size changes under therapy, it provides a good initial correspondence to extract the region of interest (ROI) around the propagated mask. To compensate for registration errors, the search region was enlarged by 50 mm in every direction to ensure that the corresponding lesion is inside the selected region and to include enough information for the U-Net (nnUNet framework). The U-Net was trained using the lymph node and soft-tissue lesions annotated in the baseline and follow-up scans of the training dataset. The validation data set was only used to monitor the training but not to select hyperparameters.

Lesion selection:

The U-Net was not constrained to segment only one lesion inside the selected region in the follow-up scan. Therefore, the lesion whose center was closest to the center of the propagated lesion was selected. To avoid annotation of wrong close-by lesions in the case of complete response, the network accepted only segmentation of lesions if the Euclidean distance of their center to the propagated lesion center was smaller than 25 mm. In cases where the network failed to segment a lesion or segmented a lesion with a diameter smaller than 5mm, an empty mask was stored that contained the information of the propagated center of gravity of the baseline lesion. Consequently, the corresponding region could be displayed by selecting the lesion. Additionally, the lesion was considered to have disappeared under therapy.

Appendix E: Details on Statistical Analysis

The statistical analysis primarily targeted two (co-primary) endpoints: reading time (seconds) and segmentation accuracy (Dice score), comparing the assisted to the manual workflow.
We considered an average Dice score loss of up to 0.05 as non-inferior. For both analyses, a Bayesian mixed effects generalized linear model was fit with the statistical software R (version 4.2.1) and the brms package (version 2.18.0). For the reading time analysis, each patient formed an observation. For the Dice analysis, the observation unit is a single lesion. The hierarchical data (dependency) structure is considered by the statistical model. For the time data, the shifted lognormal distribution is used to model the positive outcome. For the accuracy data a zero-inflated Beta regression was performed to adequately deal with the Dice score being restricted to the interval [0,1]. Adding appropriate random effects (e.g., reader, lesion within patient) to the model allows us to generalize our findings to unknown lesions, patients and readers. Weakly informative or flat prior distributions were utilized for all analyses. We quantify uncertainty with 95% posterior (quantile) credible intervals (CI). In addition, the posterior probability of each research hypothesis is reported. In a secondary analysis, inter-reader agreement was assessed by modelling the Dice score in each pair of readers in the study.

Appendix F: Inter-Reader Variability

Table F1: Intraclass correlation coefficients, comparing the reference segmentation and the manual/AI-assisted segmentations by the three readers, split by lesion size, respectively.

		**ICC**	**95% CI**
**Lesion size**	**Mode**		
all	Manual	0.80	0.76-0.83
	Assisted	0.84	0.81-0.87
<10mm	Manual	0.31	0.10-0.47
	Assisted	0.77	0.70-0.83

10-20mm	Manual	0.90	0.87-0.93
	Assisted	0.88	0.84-0.92
>20mm	Manual	0.78	0.67-0.86
	Assisted	0.82	0.73-0.89
Abbreviations: CI, confidence interval; ICC, intraclass correlation coefficient			

Appendix G: SATORI Software

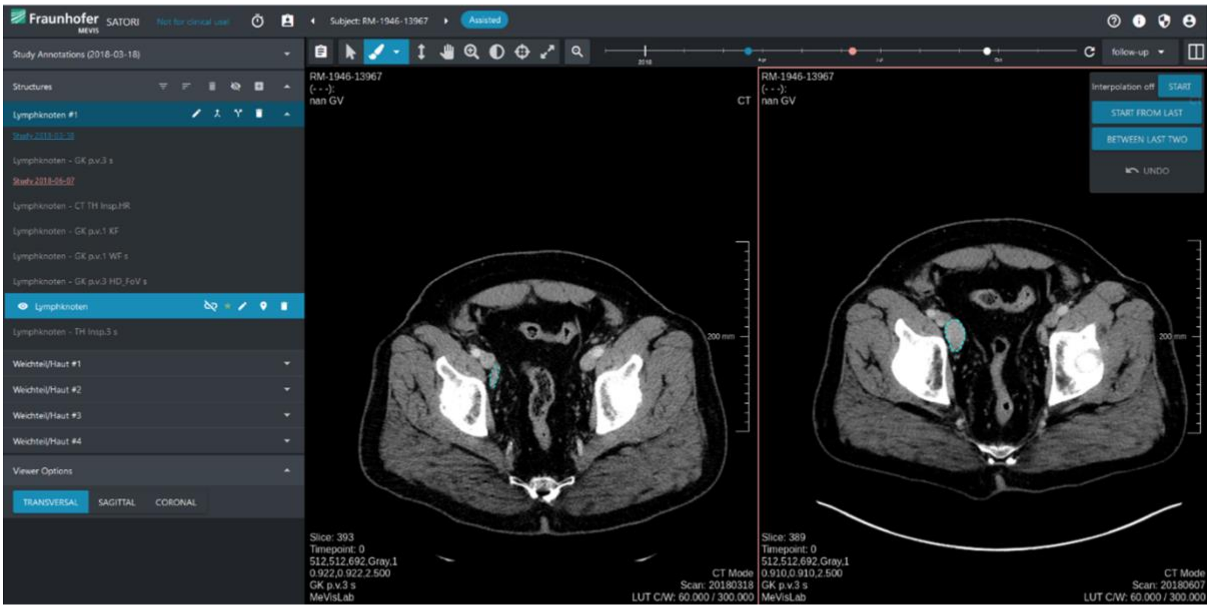

Figure G1: The custom-made reading software SATORI in the AI-assisted session. All lesions annotated on the baseline scan are listed on the left. On the baseline scan (left axial CT reconstruction), only the reference segmentation is shown (enlarged lymph node identified adjacent to the right external iliac artery, indicated by a blue oval outline). For the follow-up study (right axial CT reconstruction), for all series, an automatically computed lesion is imported, which shows progression of lesion size in comparison to the base line exam. The user can accept the lesion or manually correct it.
